# Patient and provider radiation exposure during fluoroscopy guided chemical and thermal neurolysis of genicular nerves: A prospective cohort comparison study

**DOI:** 10.1016/j.inpm.2022.100158

**Published:** 2022-11-17

**Authors:** Cole W. Cheney, Kyle P. Mele, Adrienne B. Mejia, Ankur Garg, Masaru Teramoto, Robert J. McCarthy, David R. Walega

**Affiliations:** a Division of Pain Medicine, Mayo Clinic Health Systems, Mankato, MN, USA; b Division of Pain Medicine, Northwestern University, Chicago, IL, USA; c Department of Radiology, Northwestern University, Chicago, IL, USA; d Division of Physical Medicine & Rehabilitation, University of Utah, Salt Lake City, UT, USA; e Department of Anesthesiology, Rush University, Chicago, IL, USA

## Abstract

**Purpose:**

To evaluate the differences in radiation dosimetry, fluoroscopy time and procedure time between fluoroscopy-guided chemical and thermal genicular neurolysis techniques.

**Methods:**

This single-site, open label observational cohort was done at an urban, tertiary medical center pain clinic. Board certified pain medicine physicians with at least 5 years of experience with genicular neurolysis procedures performed or supervised all interventions. Clinical characteristics and procedural details were collected at each procedure. Patients underwent chemical neurolysis using phenol or cooled radiofrequency neurolysis. Radiation dosimetry was the primary outcome and was compared between the between chemical and radiofrequency neurolysis groups.

**Results:**

Thirty-one subjects (15 had chemical and 16 had radiofrequency neurolysis procedures) underwent a total of 43 interventions. Twelve underwent bilateral procedures. Radiation dosimetry per procedure was 1.66 (0.89 to 2.45) Gy-cm^2^ for chemical and 1.76 (1.08 to 2.28) Gy-cm^2^ for radiofrequency neurolysis, adjusted mean difference −0.092 (−0.60 to 1.114, *P* ​= ​0.864) Gy-cm^2^. Procedure times were shorter for chemical compared to radiofrequency neurolysis procedures, difference 9.2 (95% CI 6.8 to 11.6, *P* ​< ​0.001) minutes; but no between treatment group differences in fluoroscopy time or interventionalist radiation exposure were observed. Higher BMI and advanced Kellgren-Lawrence grades were associated with increased patient radiation dosimetry.

**Conclusions:**

We found that patient radiation dose, fluoroscopy time, and interventionalist radiation exposure were not different between chemical and radiofrequency neurolysis. Genicular neurolysis was more rapidly performed using chemical as compared to radiofrequency neurolysis. BMI and Kellgren-Lawrence grade, but not procedural factors were associated with greater absorbed radiation doses.

## Funding

The authors have no sources of funding to declare for this manuscript.

## 1 Introduction

Knee osteoarthritis (KOA) is a common disease process afflicting 14 million people in the United States [1]. For many years, treatment was limited to conservative therapy or surgical intervention. When conservative therapy fails, a subset of patients refuse surgery or cannot be considered surgical candidates. Genicular nerve neurolysis (GN) has been used to treat KOA pain that does not improve with more conservative treatments. A traditional ablation protocol targets at least 3 genicular nerves: the superior medial (SMGN), the superior lateral (SLGN), and the inferior medial genicular nerves (IMGN) [2,3]. GN requires image guidance to identify the anatomic landmarks associated with genicular nerve location [4,5]. Common methods of GN include chemical neurolysis (ChN) and radiofrequency neurolysis (RFN) [6]. While these two approaches have not been compared directly, studies suggest similar duration of pain relief and safety profiles [7,8].

Image guidance for GN may include fluoroscopy, ultrasound or a combination thereof [9]. There is limited information on which imaging method is safer or more time efficient, but radiation exposure to patients and interventionalists from fluoroscopy is a safety concern, particularly in high volume interventional practices [7,8,10,11]. Strategies to reduce radiation intensity and fluoroscopy time are used as standard of care, and include using pulsed mode fluoroscopy (instead of continuous), image collimation, and plastic and leaded barriers [11,12]. Differences in radiation exposure with various pain procedures, including GN, are not well studied, although such data would assist providers in medical decision making and selection of imaging modality used to perform pain management procedures. The purpose of this study was to determine if there is a clinically meaningful difference in radiation exposure between genicular ChN and RFN.

## 2.1 Methods

This single-site, open label observational cohort study of patient and provider radiation exposure during fluoroscopically guided GN was approved by the Northwestern University Institutional Review Board (#STU00214249) and conducted at the Northwestern Pain Medicine Clinic. This manuscript adheres to the Strengthening the Reporting of Observational Studies in Epidemiology statement guidelines. All subjects provided informed written informed consent for study participation.

### 2.2 Study population

Eligible subjects were English-speaking adults ≤85 years of age scheduled for a GN procedure. Excluded were patients with a BMI >35 or a history of knee replacement on the ipsilateral procedure side. Patients were screened by research personnel via chart review to determine eligibility. Eligible subjects were approached for study participation and provided written informed consent prior to any study procedures.

The following patient and procedural data were collected: age (years), body mass index (BMI, kg/m^2^), sex, use of conscious sedation during the procedure, laterality of the procedure, and procedure time (seconds, defined as the duration a patient was on the procedure table after the operative knee was positioned, prepped, and draped). If a trainee was involved in the procedure, estimated percentage of trainee procedure time involvement (0–75%, 75–100%), and estimated trainee number of prior radiofrequency procedures (0–50, >50) was noted by authorized research coordinators. A musculoskeletal fellowship trained board certified radiologist recorded the severity of KOA using the Kellgren-Lawrence grading scale where grade 0 (none) equals definite absence of x-ray changes of osteoarthritis, grade 1 (doubtful) doubtful joint space narrowing and possible osteophytic lipping, grade 2 (minimal) definite osteophytes and possible joint space narrowing, grade 3 (moderate) moderate multiple osteophytes, definite narrowing of joint space, and some sclerosis and possible deformity of bone ends, or grade 4 (severe) large osteophytes, marked narrowing of joint space, severe sclerosis, and definite deformity of bone.

The interventionalist radiation exposure data was recorded by three Instadose 2® dosimeters (Mirion Technologies, Irvine, CA, USA) worn externally on the interventionalists’ lead thyroid shields. The device utilizes direct ion storage technology which, when exposed to photon radiation, leads to electron ionization. This device was linked to a Health Insurance Portability and Accountability Act (HIPPA) compliant device by Bluetooth to record the individual dosage for each unilateral procedure and the cumulative dosage to the Instadose 2® unit. The minimum reported dose and dosage range of this device is 10 ​mrem - 500 ​rem equivalent to (0.1 ​mSv - 5 Sv). Fluoroscopy time and patient radiation dosimetry were recorded by the C-arm system that was used (OEC 9900 Elite GE C-arm, General Electric Health, Inc. Chicago, Illinois). Dosimetry is indirectly estimated from a dose area product meter (product of the surface area of the patient that is exposed to the radiation at the skin entrance multiplied by the dose at this surface) and is objectively recorded in gray-centimeters squared (Gy-cm^2^) during C-arm use.

Study data were de-identified and recorded in a REDCap™ clinical database. Six attending physicians with board certification in anesthesiology and pain medicine and more than 5 years of clinical experience with neurolysis procedures performed all procedures with or without a fellow trainee.

### 2.3 Chemical neurolysis (ChN)

**Fig. 1:**
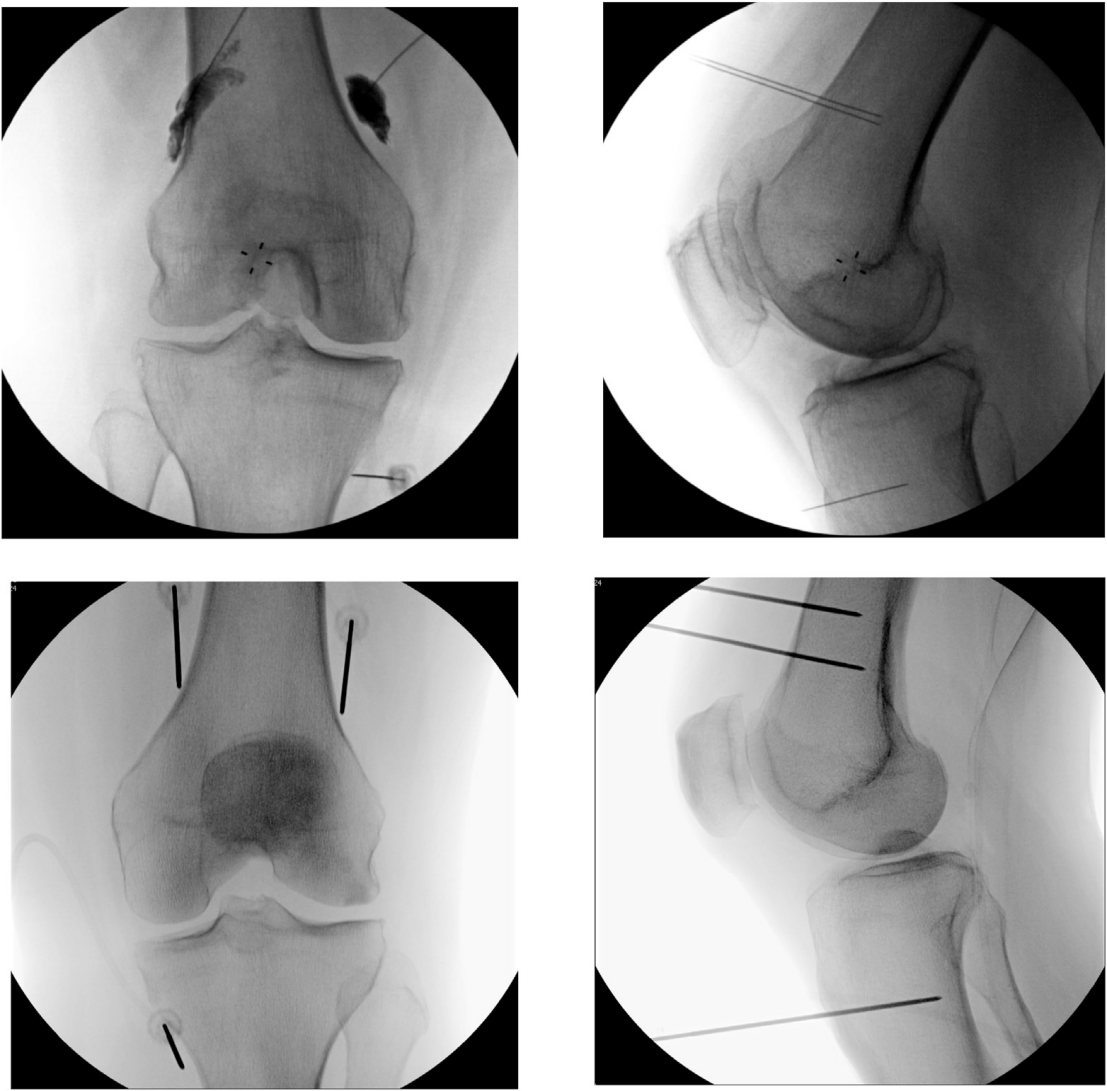
Fluoroscopic image showing final ChN needle placement in AP (UPPER LEFT) and Lateral Views (UPPER RIGHT). Final RFN needle placement in AP (LOWER LEFT) and Lateral Views (LOWER RIGHT).

The treated knee placed in 30° flexion was sterilely prepped with a solution of chlorhexidine/isopropyl alcohol and then draped with sterile towels. Fluoroscopy was then used to obtain proper anatomic location of targets for lesioning. 1–2 ​mL of 1% lidocaine was used to anesthetize the subcutaneous tissue prior to placement of three 22G 3.5-inch needles. The needles were placed at 3 positions: the junction between the lateral femoral shaft and epicondyle, the junction between the medial femoral shaft and epicondyle, and the junction between the medial tibial shaft and epicondyle. Lateral x-ray views were performed to confirm 50% depth across the femur and 75% depth across the tibia [13]. Then, 2.5 ​mL of phenol 6% 2.5 ​ml mixed with 0.5 ​ml of iohexol 300 was injected under live pulsed fluoroscopy at each location, with small movements of each needle to allow spread along the entire femoral shaft from anterior to posterior, and the posterior 1/3 of the tibial shaft [6]. The spread of contrast was evaluated in both the AP and Lateral views (Fig. 1). Needles were re-styletted and removed. Patients were observed in supine position for 30 ​min before discharge (clinic protocol to prevent injectate migration).

### 2.4 Radiofrequency neurolysis (RFN)

The treated knee placed in 30° flexion was sterilely prepped with a solution of chlorhexidine/isopropyl alcohol and then draped with sterile towels. Fluoroscopy was then used to obtain proper anatomic location of targets for lesioning. 1–2 ​mL of 1% lidocaine was used to anesthetize the subcutaneous tissue prior to placement of 17G, 75 ​mm introducer needles. The needles were placed at 3 positions: the junction between the lateral femoral shaft and epicondyle, the junction between the medial femoral shaft and epicondyle, and the junction between the medial tibial shaft and epicondyle. Lateral x-ray views were performed to confirm 50% depth across the femur and 75% depth across the tibia (Fig. 1) [13]. Then, 1–2 ​mL of lidocaine 2% was injected through each introducer prior to placing the thermocouple with 4 ​mm active tip and cooling system (Avanos, Alpharetta, Georgia) and lesioning. RFN of each of the genicular nerve was performed at 60 ​°C for 150 ​s each (maximum tissue temperature 80 ​°C). Some providers performed a second series of lesions (6 total lesions per knee).

### 2.5 Statistical analysis

Assumptions for sample size calculation were based on an evaluation of fluoroscopy times as a surrogate of radiation dose absorption during routine fluoroscopy-guided GN procedures. In a review of 84 ​GN procedures performed in 2020 (42 ChN and 42 RFN procedures) a mean difference in participant radiation exposure of 28 ​s was demonstrated. Assuming a mean fluoroscopy time of 40 ​s in the ChN group and 68 in the RFN group and a common standard deviation of 25 ​s, group sample sizes of 21 ​GN procedures would achieve 90% power to detect a difference of 28.0 between the null hypothesis mean difference of 0.0 and the actual mean difference of −28.0 ​at the 0.05 alpha using a two-sided Mann-Whitney *U* test.

The primary outcome was patient dosimetry of absorbed radiation and the primary variable of interest was the method of neurolysis (ChN vs RFN). Secondary outcomes included procedure time, fluoroscopy time, and interventionalist radiation exposure dosimetry. The distribution of the primary and secondary outcomes was evaluated using the Shapiro-Wilks test and examined graphically using q-q plots. Normally distributed continuous data are reported as mean ​± ​SD or as median (1^st^ to 3^rd^ quartile) if the assumptions of normality were not met. Univariable comparisons of the primary and secondary outcomes were compared between ChN and RFN procedure types using the Mann-Whitney *U* test. Subject characteristics and procedural data that was continuous were compared between procedure types using the Mann-Whitney *U* test. Nominal and ordinal data were compared between the groups using the Pearson chi-squared test or Fisher's Exact test. The adjusted difference in the primary outcome was determined using a generalized estimating equation with adjustment for subject or procedural variables that differed between ChN and RFN groups at *P* ​< ​0.2. Adjusted mean differences and 95% CI were calculated as a measure of the effect size.

Univariable associations of continuous patient and procedural variables with the amount of absorbed radiation was assessed using Pearson's correlation. The significance of the association of binominal subject and procedural variables with the absorbed radiation was evaluated using the Mann Whitney *U* test and the effect size of the association by calculating the Wilcoxon Mann Whitney odds. Multivariable association of factors with the amount of absorbed radiation was evaluated using a generalized estimating equation. Adjusted mean differences and 95% confidence intervals (CI) were calculated as a measure of the effect size.

All statistical tests are 2-sided. Normally distributed continuous data are presented as mean and standard deviation. Continuous data not meeting the criteria of a normal distribution are presented as median (1^st^ to 3^rd^ quartile) or range. Nominal and ordinal data are presented as n (%). Data were analyzed using RStudio version 2022.02.1 Build 461 (RStudio, PBC, Boston, MA; URL: http://www.rstudio.com/) and R version 4.1.3, release date March 10, 2022 (The R Foundation for Statistical Computing, Vienna, Austria).

## 3 Results

Thirty-one subjects who underwent 43 ​GN procedures were included in the study and subsequent analysis. Subject and procedure information is shown in Table 1.

**Table 1:** Subject characteristics and procedure details for genicular nerve chemical neurolysis (ChN) and radiofrequency neurolysis (RFN) groups.

	ChN	RFN	*P*
Number of procedures/subjects	20/15	23/16	
Age in years ​± ​SD^a^	67 ​± ​12	71 ​± ​10	0.282
Gender, n (%)^a^			
Male	3 (20)	7 (47)	0.252
Female	12 (80)	9 (53)	
Mean BMI, kg/m^2a^	27.5 ​± ​4.6	27.6 ​± ​5.0	0.993
Median (IQR) Kellgren-Lawrence grade, 0–4^b^	2 (1 to 3)	3 (3 to 3)	0.232
Trainee involvement n (%)^b^			
<75%	4 (20)	2 (9)	0.393
75–100%	16 (80)	21 (91)	
Trainee prior number of procedures, n (%)^b^			
≤50%	5 (25)	7 (30)	0.745
>50%	15 (75)	16 (70)	
Procedural sedation, n (%)^b^	10 (50)	19 (83)	0.048
Laterality n (%)^b^			
Right only	5 (25)	4 (17)	
Left only	5 (25)	5 (22)	0.752
Bilateral	10 (50)	14 (61)	
Number of lesions per knee n (%)			
3	20 (100)	18 (78)	0.051
6	0 (0)	5 (22)	
Procedure time, seconds			
Mean ​± ​SD	662 ​± ​178	1245 ​± ​316	<0.001
Median (IQR)	642 (541 to 796)	1184 (1008 to 1440)	

Data presented as mean (SD), median (1^st^ to 3^rd^ quartile), or n (%) of column. ^a^ Calculated per patient (n ​= ​31). ^b^ Calculated per knee/procedure (n ​= ​43; 20 ChN and 23 RFN).

The study sample was 10/31 (32%) male and 21/31 (67.7%) female patients, with a mean age of 69.4 ​± ​10.9 years. The mean BMI was 27.5 ​± ​4.7 ​kg/m^2^. GN procedures were performed on 21 right and 22 left knees; 24 of these procedures (12/43 patients) involved bilateral procedures performed on the same patient during the date of service. The mode of trainee number of procedures performed was greater than 50 RFN procedures, with the mode of trainee percentage contribution to procedure being 75–100%. Procedural sedation was more common during RFN procedures. All ChN procedures targeted 3 lesions per knee, whereas 22% of RFN procedures targeted 6 lesions per knee. No procedural complications occurred.

Fluoroscopy times and radiation dosimetry values are shown in Table 2.

**Table 2:** Fluoroscopy times and radiation exposure between genicular nerve chemical neurolysis (ChN) and radiofrequency neurolysis (RFN) groups.

	ChN (n ​= ​20)	RFN (n ​= ​23)	*P*
Median (IRQ) Fluoroscopy time, seconds	37.7 (21.6 to 66.8)	37.5 (31.2 to 45.6)	0.922
Median (IQR) radiation dose in Gy-cm^2^			
Per patient	2.03 (1.01 to 3.29)	2.16 (0.72 to 4.31)	0.763
Per procedure	1.66 (0.89 to 2.45)	1.76 (1.08 to 2.28)	0.845
Median (range) interventionalist radiation exposure dose in mRem	0 (0 to 10)	0 (0 to 26)	0.396

Data presented as Mean ​± ​SD or Median (IQR ​= ​1^st^ to 3^rd^ quartile) or (range ​= ​lowest to highest value).

The median (1^st^ to 3^rd^ quartile) radiation dose was 1.69 (1.01 to 2.28) Gy-cm^2^, and the median difference between the ChN and the RFN groups was −0.09 (95% CI -0.73 to 0.46, *P* ​= ​0.845) Gy-cm^2^. The mean difference adjusted for procedural sedation and procedural time was −0.09 (−0.60 to 1.114, *P* ​= ​0.864) Gy-cm^2^. Median (1^st^ to 3^rd^ quartile) fluoroscopy time for all procedures was 37.5 (24.3 to 57.4) s, and the median difference between ChN and RFN groups 0.2 (95% CI -15.8 to 18.0, *P* ​= ​0.922) seconds. The recorded interventionalist radiation exposure was below the 10 mRem lower threshold in 39/43 (91%) of the procedures. During only 4 procedures was the interventionalist exposure above the 10mRem threshold, the highest exposure was 26 mRem in one case. There was not a statistically significant difference in interventionalist radiation exposure between ChN and RFN groups.

Binominal variables associated with the procedural radiation dosimetry are shown in Table 3 and continuous variables in Table 4.

**Table 3:** Association of binomial variables with procedural radiation dosimetry.

Procedural radiation dose in Gy-cm^2^				
Variable	Positive	Negative	WMWodds (95% confidence interval)	*P*
Female gender	1.64 (0.93 to 2.10)	1.89 (0.99 to 3.65)	0.73 (0.36 to 1.53)	0.422
Kellgren-Lawrence grade >2	2.03 (1.63 to 2.69)	1.01 (0.67 to 1.76)	3.13 (1.39 to 6.71)	0.005
Conscious sedation	1.85 (1.26 to 2.28)	1.64 (0.83 to 3.18)	1.31 (0.63 to 2.68)	0.484
Left laterality	1.66 (0.97 to 2.34)	1.85 (0.97 to 2.31)	0.92 (0.47 to 1.81)	0.808
Three lesions treated	1.66 (1.06 to 2.14)	2.34 (0.61 to 3.65)	1.24 (0.44 to 3.41)	0.705

Data presented as median (1^st^ to 3^rd^ quartile), WMW = Wilcoxon-Mann-Whitney odds.

**Table 4:** Association of interval patient and procedural variables with radiation dosimetry.

Variable	Pearson Correlation coefficient	95% Confidence Intervals	*P*
Age	0.177	−0.132 to 0.452	0.778
BMI	0.368	0.072 to 0.599	0.015
Fluoroscopy time	0.128	−0.181 to 0.411	0.414
Procedure time	−0.007	−0.306 to 0.294	0.967

The only binominal factor associated with an increased odds of increased procedural radiation was a Kellgren-Lawrence grade >2, WMW odds 3.13 (95% CI 1.39 to 6.71, *P* ​= ​0.005). The only interval variable associated with an increase in patient radiation dosimetry was BMI (Fig. 1).

## 4 Discussion

This study of GN procedures demonstrates that radiation dose, fluoroscopy time, and interventionalist radiation exposure were not statistically different between procedures. Procedure time was consistently lower during ChN when compared to RFN. Procedure time differences are not surprising. Neurolysis time is far shorter for ChN than cooled RFN, due to cooled RFN priming and ablation duration, the latter being 150 ​s. As numerous studies now recommend additional neural targets for RFN to optimize outcomes, RFN procedure time will be further prolonged when these new targets are universally accepted as the standard of practice [13,14]. In theory, ChN allows a wider surface area in which to capture numerous sensory afferent targets that are only captured with RFN when additional time-based lesioning is performed. This study evaluated procedure time only; post-procedure care varied greatly between techniques and warrants further study.

While this study's RFN protocol targeted the SMGN, SLGN, and IMGN as classically reported [[2], [3], [4]], recent recommendations include additional targets of the nerves to vastus medialis, vastus lateralis, and vastus intermedius and an additional lesion of the SLGN [[15], [16], [17]]. This would require an additional 2 cycles of 150 ​s to ablate all of these targets, adding a minimum of 5 ​min to this procedure.

We found BMI and higher Kellgren-Lawrence grade to be associated with greater radiation doses (Table 3, Table 4). A positive relationship between BMI and radiation dose was expected as fluoroscopy systems auto titrate radiation exposure to obtain an image depending on tissue density as demonstrated in previous studies [18]. A positive relationship between Kellgren-Lawrence grade and radiation dose is a novel finding, though this was likely mediated by the known positive relationship between higher BMI and progression of knee osteoarthritis [19].

The overall radiation dose of each procedure was 1.66 and 1.76 Gy-cm^2^ DAP for ChN and RFN, respectively, and the difference was not statistically different despite longer procedure time, a finding consistent with previous literature [18]. The Society of Interventional Radiology – Cardiovascular and Interventional Radiology Society of Europe (SIR-CIRSE) recommends a threshold DAP of 500 Gy-cm^2^ [20]. ChN and RFN would account for 0.003% and 0.004%, respectively, of a patient's maximum yearly cumulative dose. The United States Nuclear Regulatory Commission (USNRC) recommends a maximum yearly medical worker exposure dose of 5,000 ​mrem and 30,000 ​mrem per year to the whole body and thyroid gland, respectively. [21]. The worst single interventionalist exposure (26 ​mrem) would account for 0.005% and 0.0009% of the interventionalist's maximum yearly cumulative dose for whole body and thyroid gland, respectively.

Limitations of this investigation must be acknowledged. RFN was performed using cooled RF which requires longer lesioning times to obtain ideally sized lesions, as compared to conventional RF which requires typically shorter lesion times at the expense of smaller lesion sizes that are more apt to “miss” the sensory nerve target. Therefore, our methods of GN may not mirror universal practice patterns. Newer protocols involve RF systems with larger gauge size, multiple tines, and multiple lesions to increase lesion size. Larger gauge size and multiple tines increase lesion size without prolonging lesion time. In addition, our sample size calculation was based on data from 84 consecutive ChN and RFN procedures from a single provider who performs six RFN lesions as the practice standard, but the study was performed with several physicians who continue to perform RFN at the 3 sites classically described in prior clinical trials. This operational difference may have resulted in a falsely large outcome difference used to calculate our sample size calculation, and a larger study may show more significant group differences. In addition, variance between study RFN techniques resulted in cumulative outcomes based on a non-uniform protocol. Finally, fellow trainees were involved in the conduct of these GN procedures under direct supervision by experienced faculty, and although we accounted for fellow experience and amount of involvement in the respective neurolysis procedures, this may have created variance in procedure times.

## 5 Conclusions

Genicular neurolysis can be more rapidly performed using ChN compared to RFN. Other outcomes including patient radiation dose, fluoroscopy time, and interventionalist radiation exposure were not different when comparing ChN and RFN. We found BMI and Kellgren-Lawrence grade to be associated with greater absorbed radiation dose; other patient and procedural factors were not associated with greater radiation doses. Optimizing procedural efficiencies and treatment outcomes should continue to be balanced with safety for patients as well as providers as future studies are performed.

## Declaration of competing interest

The authors declare that they have no known competing financial interests or personal relationships that could have appeared to influence the work reported in this paper.
